# FABP1 controls hepatic transport and biotransformation of Δ^9^-THC

**DOI:** 10.1038/s41598-019-44108-3

**Published:** 2019-05-20

**Authors:** Matthew W. Elmes, Lauren E. Prentis, Luke L. McGoldrick, Christopher J. Giuliano, Joseph M. Sweeney, Olivia M. Joseph, Joyce Che, Gregory S. Carbonetti, Keith Studholme, Dale G. Deutsch, Robert C. Rizzo, Steven E. Glynn, Martin Kaczocha

**Affiliations:** 1 Department of Biochemistry and Cell Biology, Stony Brook University, Stony Brook, New York, 11794 USA; 2 Department of Anesthesiology, Stony Brook University, Stony Brook, New York, 11794 USA; 3 Graduate Program in Molecular and Cellular Biology, Stony Brook University, Stony Brook, New York, 11794 USA; 4 Department of Applied Mathematics and Statistics, Stony Brook University, Stony Brook, New York, 11794 USA

**Keywords:** Protein transport, Mechanism of action, Molecular modelling, X-ray crystallography

## Abstract

The increasing use of medical marijuana highlights the importance of developing a better understanding of cannabinoid metabolism. Phytocannabinoids, including ∆^9^-tetrahydrocannabinol (THC), are metabolized and inactivated by cytochrome P450 enzymes primarily within the liver. The lipophilic nature of cannabinoids necessitates mechanism(s) to facilitate their intracellular transport to metabolic enzymes. Here, we test the central hypothesis that liver-type fatty acid binding protein (FABP1) mediates phytocannabinoid transport and subsequent inactivation. Using X-ray crystallography, molecular modeling, and *in vitro* binding approaches we demonstrate that FABP1 accommodates one molecule of THC within its ligand binding pocket. Consistent with its role as a THC carrier, biotransformation of THC was reduced in primary hepatocytes obtained from FABP1-knockout (FABP1-KO) mice. Compared to their wild-type littermates, administration of THC to male and female FABP1-KO mice potentiated the physiological and behavioral effects of THC. The stark pharmacodynamic differences were confirmed upon pharmacokinetic analyses which revealed that FABP1-KO mice exhibit reduced rates of THC biotransformation. Collectively, these data position FABP1 as a hepatic THC transport protein and a critical mediator of cannabinoid inactivation. Since commonly used medications bind to FABP1 with comparable affinities to THC, our results further suggest that FABP1 could serve a previously unrecognized site of drug-drug interactions.

## Introduction

The clinical use of cannabinoids has shown great promise in treating a multitude of pathologies including epilepsy, glaucoma, chronic pain, multiple sclerosis, and chemotherapy-induced nausea^1–3^. ∆^9^-Tetrahydrocannabinol (THC), through activation of type-1 cannabinoid receptors (CB_1_), is principally responsible for the pharmacological and psychoactive effects elicited by the consumption of marijuana. However, THC metabolites and other prominent phytocannabinoids such as cannabidiol (CBD) also contribute to the complex pharmacology of cannabis. The stark increase in recreational and medical marijuana use in recent years highlights the importance of developing a more thorough understanding of cannabinoid metabolism and predicting potential sites of drug-drug-interactions with other commonly prescribed medications.

**Figure 1:**
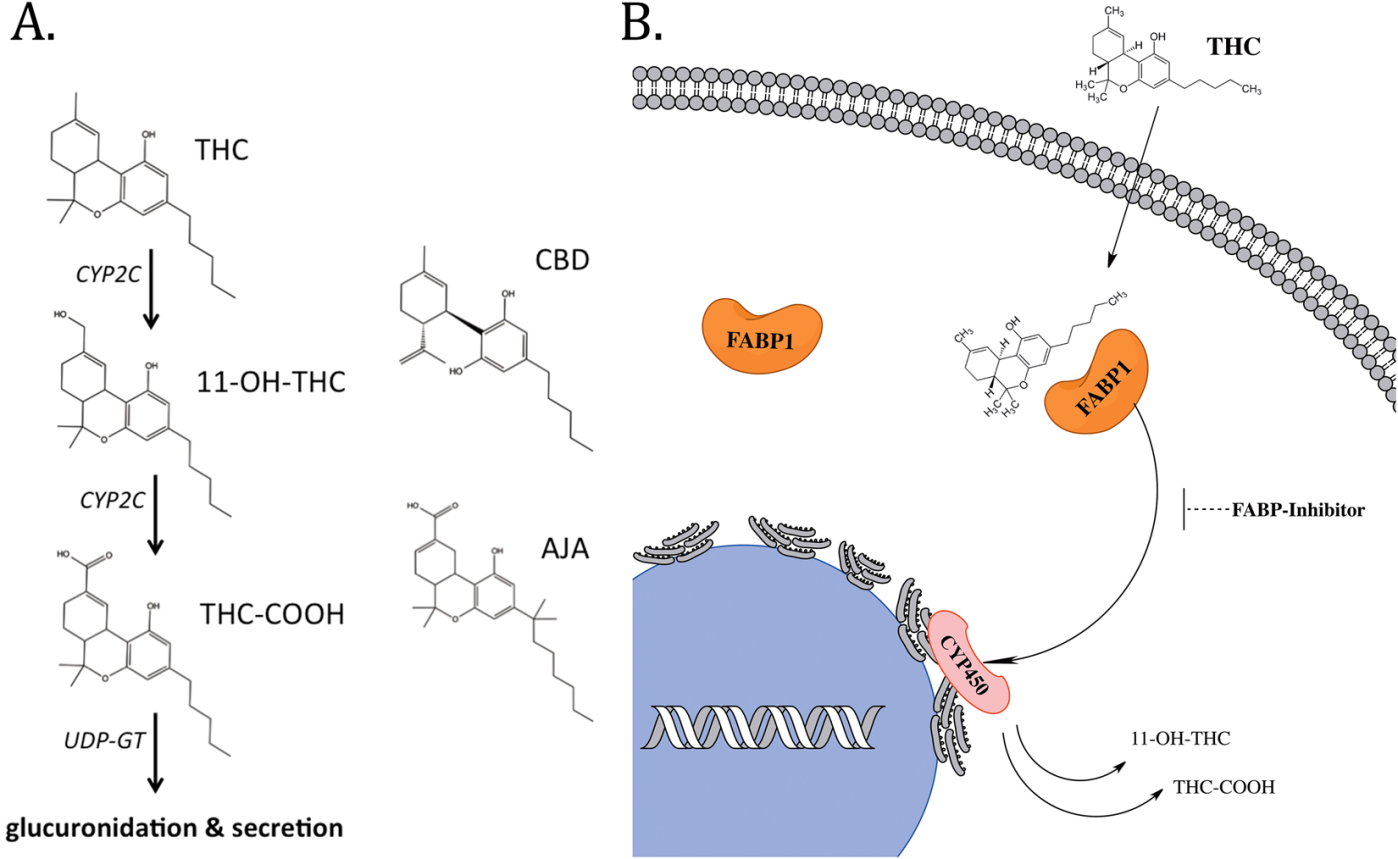
Overview of the major THC metabolic pathway. (**A**) Chemical structures of relevant cannabinoids and CYP2C-generated THC metabolites. (**B**) Schematic of FABP1-mediated transport of THC for subsequent metabolism by intracellular CYP450 enzymes.

Metabolism of THC occurs primarily within the liver by hydroxylation and oxidation reactions catalyzed the cytochrome P450 (CYP450) enzymes; with members of the CYP450-2C subfamily of isoenzymes playing the major role in rodents and humans^4,5^. Phase I metabolism involves hydroxylation of THC to its primary metabolite 11-hydroxy-Δ^9^-tetrahydrocannabinol (11-OH-THC). This metabolite retains high CB_1_ receptor affinity and may even be slightly more psychoactive than the parent THC compound itself^6^. 11-OH-THC in turn undergoes further oxidation by CYP450s to the inactive secondary metabolite 11-nor-9-carboxy-Δ^9^-tetrahydrocannabinol (THC-COOH) (see Fig. 1A for THC metabolic pathway schematic). Phase II metabolism involved glucuronidation of THC-COOH by UDP-glucuronoyltransferases (UDP-GT), conferring sufficient aqueous solubility for bioelimination through urine, sweat, and feces^7^. The CYP450s and UDP-GTs are localized to the endoplasmic reticulum of hepatocytes^8,9^. The highly lipophilic nature of cannabinoids necessitates the existence of a mechanism(s) to facilitate their cytoplasmic transport to intracellular metabolic enzymes.

A recent report from our group demonstrated that brain-expressed fatty acid-binding proteins (FABPs) can function as intracellular carriers for THC and CBD^10^. However, mammals express ten unique FABP isoforms with distinct tissue-specific expression patterns, and none of the FABP subtypes that are present in the brain are also found in liver^11^. Two members of the FABP family are found in hepatic cells; liver-type FABP (FABP1) and intestinal-type FABP (FABP2). We consider FABP1 to be the more likely candidate to facilitate cytoplasmic THC trafficking due to its high expression levels in hepatocytes (2–10% of total cytosolic protein^12–14^) and its ability to bind a wide range of structurally diverse lipophilic ligands, including endocannabinoids and synthetic cannabinoids^15–18^. FABP2 is found at only trace levels in liver and could play a secondary role in facilitating phytocannabinoid transport^19,20^. Recently mouse FABP1 was shown to interact with phytocannabinoids *in vitro* with high affinities^16^. Furthermore, THC treatment induces hepatocyte accumulation of endocannabinoids in an FABP1-dependent manner, likely indicating competition for FABP1-mediated cellular uptake^21^. Here, we demonstrate that FABP1 plays a major role in governing THC biotransformation and metabolism by facilitating its cytoplasmic transport to hepatic CYP450 enzymes (see Fig. 1B for schematic).

## Results

### Binding of cannabinoids to FABP1

**Figure 2:**
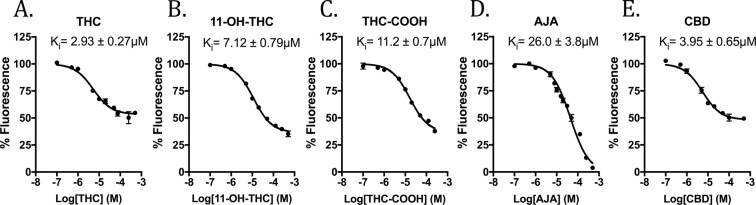
Binding of cannabinoids to FABP1. DAUDA displacement curves and Ki values for (**A**) THC, (**B**) 11-OH-THC, (**C**) THC-COOH, (**D**) AJA, and (**E**) CBD are shown.

Binding affinities of phytocannabinoids to FABP1 were assessed by DAUDA displacement from the FABP1 binding pocket^22^. This environment-sensitive fatty acid analog alters its fluorescence intensity and emission spectra when in a hydrophobic environment, such as within the FABP binding pocket. Binding affinity of the DAUDA probe to FABP1 was determined in house by saturation binding assays (K_d_ = 1.07 ± 0.17 µM) and was found to be in close agreement with previously published data^23^. THC and CBD were found to interact with FABP1 with moderately strong affinities (K_i_ = 2.93 ± 0.27 µM and 3.95 ± 0.65 µM, respectively) (Fig. 2A,E). The low micromolar FABP1 affinities of these phytocannabinoids are similar to those of the brain-expressed FABP isoforms (i.e. FABP3, FABP5, and FABP7)^10^. FABP1 is unique among the FABP family because its large binding cavity confers the ability to bind two fatty acid ligands simultaneously^24^. Our binding studies indicate that THC and CBD maximally displace nearly 50% of the fluorescent probe, as has been previously demonstrated with the mouse homolog, suggesting that they occupy only one FABP1 binding site^16^. Co-incubation of THC with CBD did not further reduce DUADA fluorescence intensity, suggesting that these phytocannabinoids bind to the same region of the FABP1 binding pocket (Supplementary Fig. S1). The physiological FABP1 ligand oleic acid was used for assay validation and was found to nearly completely displace DAUDA fluorescence at 10 µM (96.5 ± 0.15% displacement), consistent with previous reports demonstrating that it binds to both sites within FABP1 with high affinity^25^. Palmitoylethanolamide (PEA), a fatty acid amide reported to exhibit little or no affinity for FABP1 *in vitro*^16^, did not produce any reduction in fluorescence intensity (101.8 ± 3.3% fluorescence at 50 µM PEA) providing further validation to our assay (Supplementary Fig. S1).

We additionally explored FABP1 binding to the major primary and secondary THC metabolites, 11-OH-THC and THC-COOH. These metabolites were each found to bind FABP1 with somewhat reduced affinities compared to the parent THC compound (K_i_ = 7.12 ± 0.79 µM and 11.2 ± 0.7 µM, respectively) (Fig. 2B,C). Lastly, we explored the binding of ajulemic acid (AJA), a non-psychoactive synthetic THC-COOH analog. We observed relatively weaker binding of this compound to FABP1 (K_i_ = 26.0 ± 3.8 µM) (Fig. 2D). Interestingly, AJA was able to fully displace DAUDA from the FABP1 binding pocket in this concentration range, unlike the other cannabinoids and THC metabolites (Fig. 2). Prior to glucuronidation, the THC metabolites are not sufficiently hydrophilic for normal bodily excretion^7^. Our data demonstrating moderately high affinities of THC metabolites to FABP1 suggest that it may also mediate the transport of the primary and secondary THC metabolites to and from their respective metabolic enzymes, the CYP450s and UDP-GTs.

### Structural interactions of THC with FABP1

**Figure 3:**
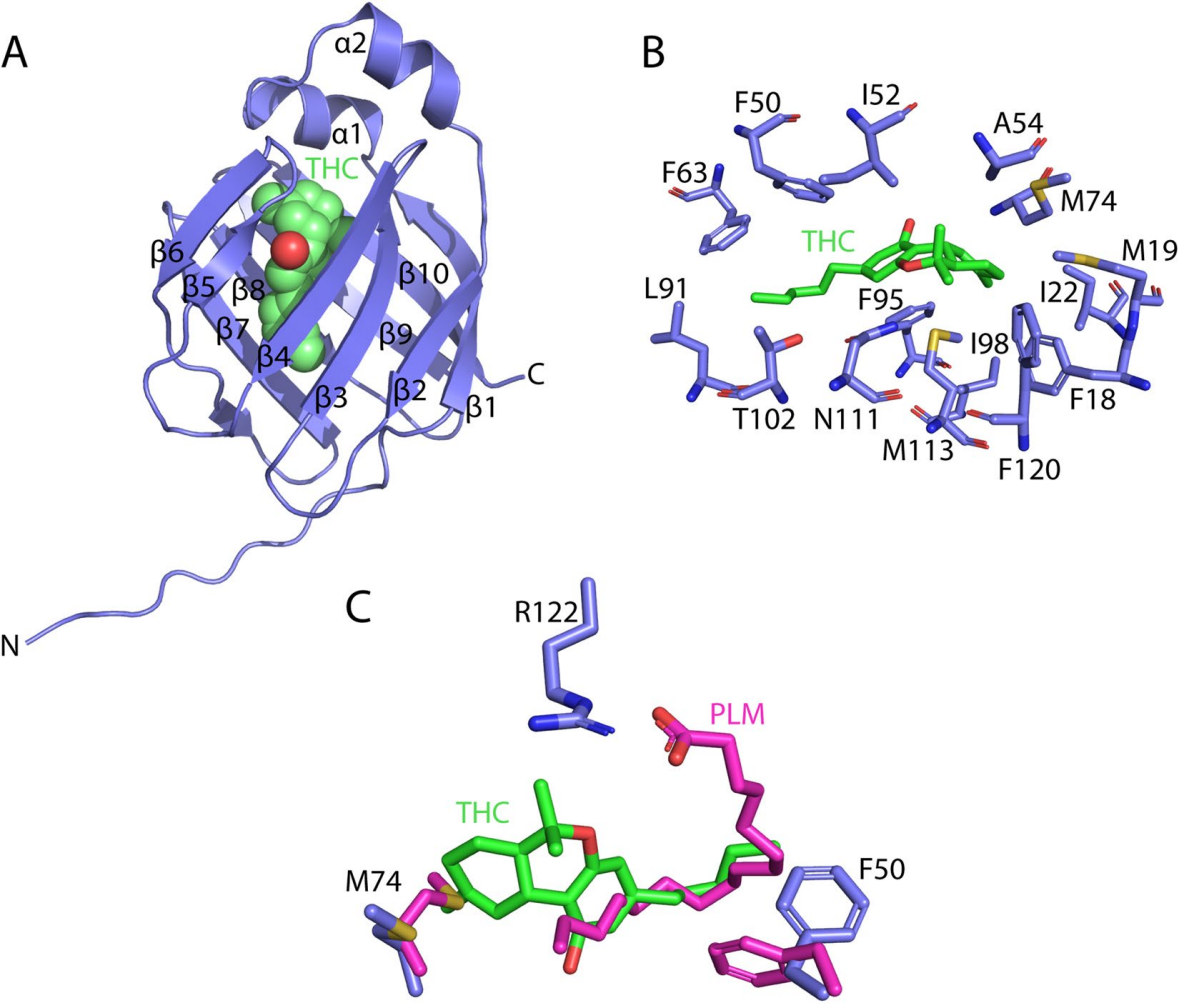
Structure of THC bound to FABP1. (**A**) Structure of FABP1 containing a single molecule of THC within the binding cavity (PDB: 6MP4). (**B**) Structure of the THC binding site showing side chains for residues positioned within 4.5 Å. Density surrounding THC is shown from a simulated annealing 2m*F*_o_ − D*F*_c_ OMIT map contoured at 0.8 σ. (**C**) Structural overlay of THC and palmitic acid (PDB: 3STM) bound to FABP1 showing the distinct binding sites of the molecules with altered positions of Met74 and Phe50.

To reveal the precise interactions used by FABP1 to bind THC, we determined the crystal structure of *N*-terminally His-tagged human FABP1 after delipidation followed by overnight incubation in the presence of 350 μM THC dissolved in DMSO (PDB: 6MP4, Supplementary Table S1). The resulting crystals diffracted to 2.5 Å and belonged to a near identical space group to previously determined structures of FABP1 bound to palmitic acid (PDB: 3STM)^26^. The structure was solved by molecular replacement using the palmitic acid bound structure as a search model with eight copies of FABP1 present in the crystal asymmetric unit. Examination of the m*F*_o_ − D*F*_c_ electron density maps after initial structure refinement revealed the presence of density in the binding cavities of three FABP1 molecules that could be modeled as THC (Fig. 3A). The density was weakest for the cyclohexene ring possibly resulting from alternative conformations of this ring in the structure. Accordingly, simulated annealing OMIT maps were calculated to confirm the presence of bound THC in these molecules (Fig. 3B). Weaker density was observed but not modeled in the binding pockets of the other FABP1 copies, likely representing lower occupancy THC molecules resulting from the poor solubility of THC in aqueous solution. Examination of the THC binding site revealed that the conjugated rings of the cannabinoid occupy a hydrophobic pocket within the FABP1 cavity lined by the sidechains of Phe18, Met19, Ile22, Ile52, Met74, Phe95, Phe98, Met113, and Phe120. Additionally, the O1 atom of the pyran ring is well placed to form a hydrogen bond with Asn111. The hydrocarbon tail of THC extends out towards the pocket entrance close to the sidechains of Phe50, Ile91, Thr102 and Phe93. Importantly, the bulky THC molecule occupies the majority of the FABP1 binding cavity consistent with the binding of a single cannabinoid molecule observed in our fluorescence measurements.

A comparison of our THC bound structure to FABP1 in complex with palmitic acid reveals that THC occupies a largely non-polar pocket some distance from the more polar environment of the fatty acid (Fig. 3C). For example, the ionic interaction formed between the negatively charged head group of the fatty acid and the sidechain of Arg122 is absent in our structure and is instead satisfied by a malonate ion originating from the crystallization solution. The sidechain of Met74 is significantly repositioned to accommodate the large THC rings in the hydrophobic pocket and the sidechain of Phe50 rotates to pack against the short hydrocarbon tail of THC. Intriguingly, the β-turn connecting strands β3 and β4 undergoes a large movement that closes the residues of the turn onto the THC molecule and brings Ala54 into hydrophobic contact distance, thereby closing off the FABP1 ligand entry portal, which is not observed in the palmitic acid-bound structure (Supplementary Fig. S2). THC localization within the binding pocket compared to ligand poses from published fatty acid-bound human FABP1 structures unveil considerable steric clashes^26,27^, suggesting that non-competitive co-binding of THC with endogenous lipids is unlikely (Fig. 3C and Supplementary Fig. S2).

### *In silico* predictive modeling

**Figure 4:**
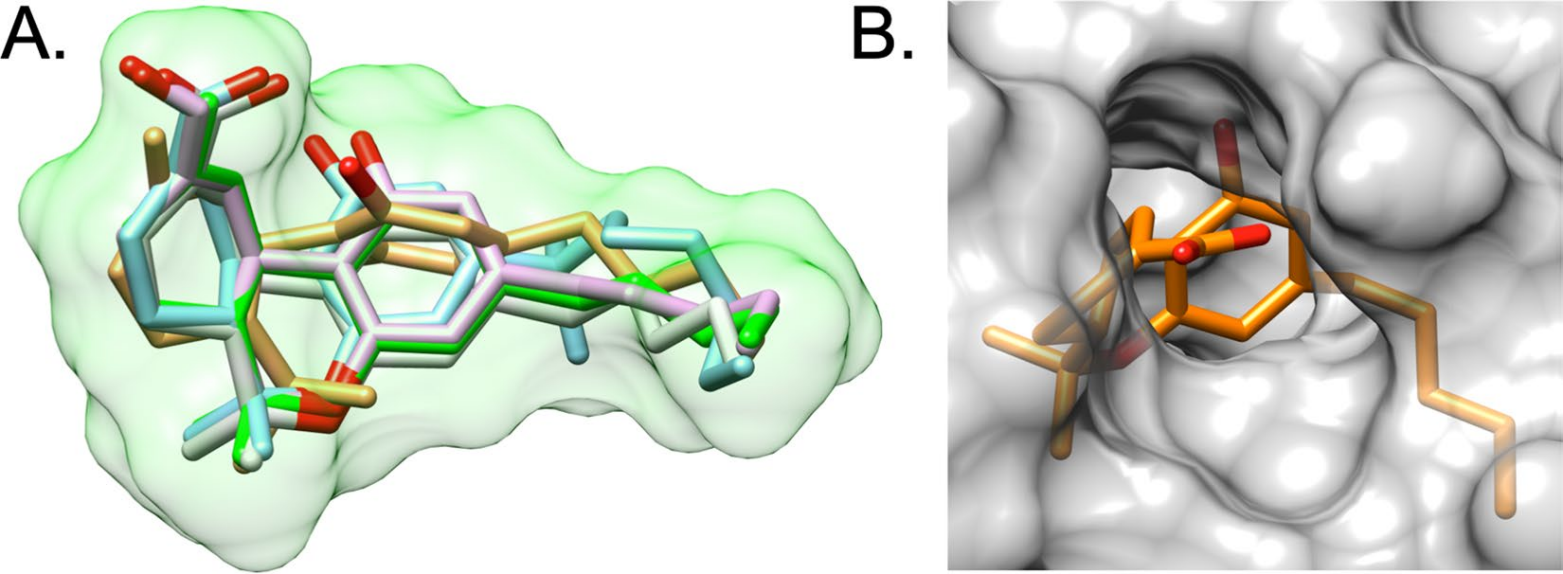
Computational modeling. (**A**) Overlay of docked poses for THC (green), 11-OH-THC (pink), THC-COOH (light green), AJA (blue), and CBD (tan). Green molecular surface for THC. Protein residues hidden for clarity. (**B**) Molecular surface of FABP1 zoomed in on the metabolite THC-COOH highlighting solvent exposure of the carboxylic acid moiety.

Based on the binding pose observed for THC in the X-ray structure, we used the program DOCK^28^ to computationally predict binding geometries of the primary THC metabolite 11-OH-THC, secondary THC metabolite THC-COOH, the THC isomer CBD, and the cannabinoid analog AJA. The objective was to assess if these cannabinoids and metabolites would be well-accommodated in the FABP1 binding site and to determine whether computed interaction energies would correlate with the experimental trends. From a geometric perspective (Fig. 4A), the best-docked pose for each ligand showed well-overlapped conformations with the sole exception of CBD which required a slight scaffold rotation to alleviate steric clashes arising from breaking the cyclic ether bond. Interestingly, from an energetic perspective (Supplementary Table S2), the standard DOCK grid energy scores (van der Waals plus electrostatic energy) are negatively correlated with the experimentally-determined activity (Supplementary Fig. S3). Further inspection revealed that the increase in scores, relative to THC, was dominated by favorable increases (Supplementary Fig. S3) in van der Waals packing (kcal/mol), especially for the closely related series that tracked with the concomitant increase in size (MW) going from THC (VDW = −54.41, MW = 314.47) > 11-OH-THC (VDW = −56.50, MW = 330.47) > THC-COOH (VDW = −59.13, MW = 343.44) > AJA (VDW = −64.31, MW = 399.55).

Recognizing that the geometric changes for the series THC > 11-OH-THC > THC-COOH involve increasingly polar character, and that the standard DOCK grid score only crudely accounts for solvation, for comparison, we recomputed the energy scores using the more sophisticated MM-GBSA scoring function (Hawkins *et al*.^29,30^ Generalized Born model) that includes more accurate desolvation penalties for the ligand, protein, and complex, which can formally be expressed as ΔG’s of hydration for each species. As shown in Supplementary Table S2 and Fig. S3, the computed MM-GBSA scores agree remarkably well with the overall trends in the experimental data, with the exception of CBD for which the score (−32.76 kcal/mol) is over-predicted relative to THC (−25.44 kcal/mol) despite THC displaying marginally greater activity. Particularly interesting is the fact that the ΔG hydration values for the ligand alone track well the experimental activities going from most to least potent THC (−6.79 kcal/mol) > CBD (−9.22 kcal/mol) > 11-OH-THC (−14 kcal/mol) > THC-COOH (−85.67 kcal/mol) > AJA (−85.13 kcal/mol). Thus, it is reasonable to propose that affinity for FABP1 is highly influenced by the polarity of the ligand. As the polarity of the ligand increases, the stronger the desolvation penalty and weaker overall binding within the hydrophobic pocket. This hypothesis also makes sense in light of the fact that the -OH and -COOH groups on the metabolites are pointing out of a solvent-exposed region near the β-turn connecting strands β5 and β6, whereby they could interact more strongly with aqueous solvent (Fig. 4B).

### *Ex vivo* THC biotransformation by primary mouse hepatocytes

**Figure 5:**
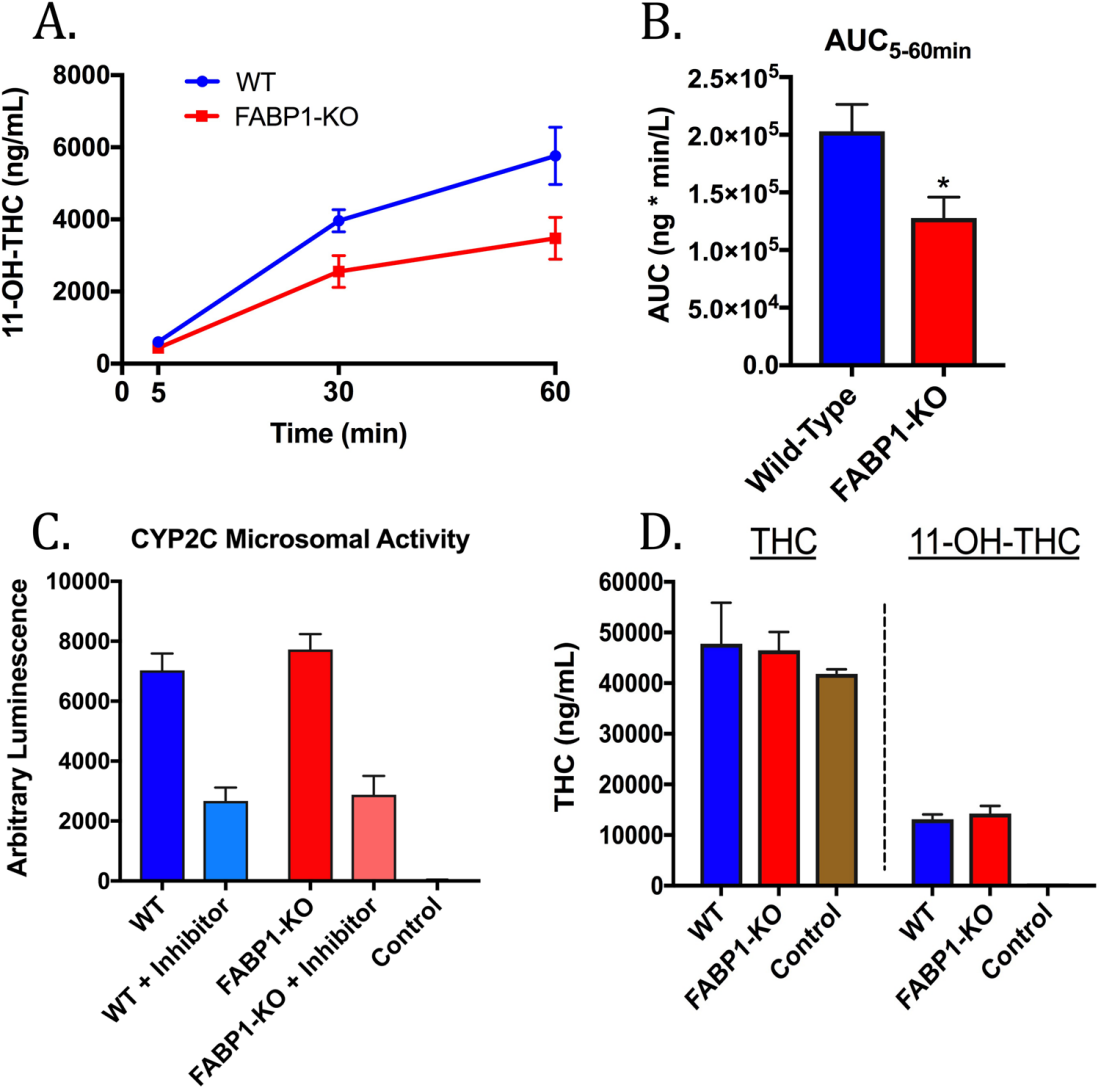
THC biotransformation by primary mouse hepatocytes. (**A**) Time-course of *ex vivo* THC biotransformation to 11-OH-THC in hepatocytes derived from WT and FABP1-KO mice. (**B**) 5–60 minute AUC analysis of 11-OH-THC formation by cultured primary hepatocytes. (**C**) *In vitro* conversion of the luciferin-H substrate by crude hepatic microsomes or BSA control in the presence or absence of the CYP2C9 inhibitor sulphaphenazole (30 µM). (**D**) *In vitro* THC biotransformation by hepatic microsomes or by BSA control. *p < 0.05 by t-test.

To determine whether FABP1 regulates THC metabolism, primary hepatocytes were isolated from WT and FABP1-KO mice and incubated with THC (10 µM) for 5, 30, or 60 minutes. This concentration and incubation time were selected based on previous THC treatment of cultured hepatic cells^21,31^. At the indicated time points the cells and culture media were harvested and levels of the THC metabolite 11-OH-THC were quantified (Fig. 5A). Area under the curve (AUC) of the resulting 11-OH-THC graph shows a significant difference between the WT and FABP1-KO groups (Fig. 5B), confirming reduced 11-OH-THC production in FABP1-KO hepatocytes. Microsomal preparations from WT and FABP1-KO mice showed similar overall activity and inhibition by the CYP2C9 inhibitor sulphaphenazole, suggesting little or no difference in CYP2C activity between the genotypes (Fig. 5C). Lastly, crude microsomes isolated from WT and FABP1-KO hepatocytes were incubated with THC. Our results demonstrate similar levels of 11-OH-THC production between the genotypes (Fig. 5D), confirming that the lower rate of THC biotransformation observed in FABP1-KO hepatocytes cannot be attributed to inherent differences in CYP450 activity between WT and FABP1-KO mice, and instead suggest a defect in intracellular THC transport to CYP450 enzymes in FABP1-KO hepatocytes.

### Comparative THC pharmacokinetics

The pharmacokinetics of THC were subsequently examined to determine whether FABP1 regulates THC biotransformation *in vivo*. Wild-type and FABP1-KO mice were administered a single dose of THC (10 mg/kg, i.p.) and blood plasma and brains were harvested at several time points during a 24-hour time course (0, 0.5, 1, 2, 3, 4, 6, 7, and 24 hours post-THC). Levels of THC and its major primary and secondary metabolites (11-OH-THC and THC-COOH) were subsequently quantified by LC-MS/MS.

THC levels were significantly elevated in FABP1-KO brains (p = 0.0012) compared to WT, whereas only a corresponding trend was observed in plasma levels (p = 0.1219) (Table 1, Supplementary Fig. S4). Total 11-OH-THC levels were similar between the genotypes, though this metabolite remained at detectable levels for 1–2 hours longer in FABP1-KO brain and plasma than in the wild-type animals (Table 1 and Supplementary Fig. S4). Maximal levels of the secondary THC metabolite THC-COOH were observed at later time points in FABP1-KO brain (∆t_max_ = 4 hours), but was found to be similar in plasma (Supplementary Fig. S4). FABP1-KO animals exhibited reduced rates of THC biotransformation, as indicated by the ~6-fold increase in the brain AUC compared to the WT groups (Table 1). Consistent with this, comparison of THC-COOH/THC ratios (AUC_THC-COOH_/AUC_THC_) revealed a ~3–4 fold lower ratio in FABP1-KO brains (Table 1). These pharmacokinetic data confirm that FABP1-KO mice exhibit reduced metabolism of THC compared to their WT counterparts, consistent with lower THC biotransformation observed in FABP1-KO hepatocytes *in vitro* (Fig. 5).

**Table 1:** Pharmacokinetic data for THC.

		THC		11-OH-THC			THC-COOH		
		AUC	C_max_	AUC	C_max_	M/P	AUC	C_max_	M/P
Brain	**Wild-type**	1446 ± 411.7	531.0 ± 71.35	1327 ± 329.3	484.4 ± 207.8	0.918	362.8 ± 36.94	58.33 ± 1.21	0.251
	**FABP1-KO**	7363 ± 949.0*	881.9 ± 574.1	1244 ± 314.6	243.6 ± 114.3*	0.169	522.6 ± 194.4	50.02 ± 3.16*	0.071
Plasma	**Wild-type**	11497 ± 2077	13575 ± 2615	59.41 ± 14.58	29.46 ± 6.17	0.0052	506.3 ± 51.35	87.12 ± 5.21	0.0440
	**FABP1-KO**	25220 ± 7334	34753 ± 2005*	111.9 ± 18.37	50.63 ± 11.82	0.0044	543.7 ± 21.17	78.68 ± 2.26	0.0215

AUC: brain (pmol*h/g), plasma (µg*h/L). C_max_: brain (pmol/g), plasma (ng/mL). M/P: metabolite to parent (THC) ratio (AUC_m_/AUC_p_). *p < 0.05 by t-test compared to the respective WT group.

### Comparative THC pharmacodynamics

Our data establish that FABP1 regulates THC biotransformation *in vitro* and *in vivo*. We next assessed whether these pharmacokinetic differences are reflected as enhanced and/or prolonged cannabimimetic effects of THC in FABP1-KO mice. CB_1_ receptor agonists induce a series of behavioral effects termed the ‘cannabinoid tetrad’^32^. The classical tetrad is characterized by hypothermia, antinociception, catalepsy, and reduced spontaneous locomotor activity. THC is well documented to produce significant effects in all facets of the tetrad in a CB_1_ receptor-dependent manner^33^.

Measurements of body temperature and spontaneous activity were selected as readouts of THC effects as they allow for quantification of THC’s physiological effects in an objective, robust, and real-time manner. Hypothermia and hypomotility models also hold inherent advantages over other tetrad tests (i.e. analgesia and catalepsy) in that they allow for repeated measures necessary for kinetic analysis without risk of desensitization of the animals to the experimental conditions. Body temperature was measured using iButton Thermocrons that permit nearly continuous core temperature readings. Locomotor activity was measured by a photobeam-activity home cage system.

Hypothermia and spontaneous locomotor activity were assessed over a 12 hour period following a single intraperitoneal (i.p.) injection of 10 mg/kg THC or vehicle (1:1:18 DMSO:Cremophor-EL:saline) to naïve mice. This dose was selected because it produces submaximal cannabimimetic effects and potentiation of THC effects is unlikely to be hampered by a ceiling effect^33,34^. All mice were kept on a 12/12 hour light/dark cycle and THC was administered immediately prior to the dark phase to control for circadian effects. Gender has been reported to influence some phytocannabinoid-induced behavioral effects in rodents, though notably, the vast majority of these studies were performed in rats and to our knowledge no direct male-to-female comparisons have yet been demonstrated in mice^35–38^. Therefore, in the present study THC pharmacodynamics were independently assessed in mice of both genders.

**Figure 6:**
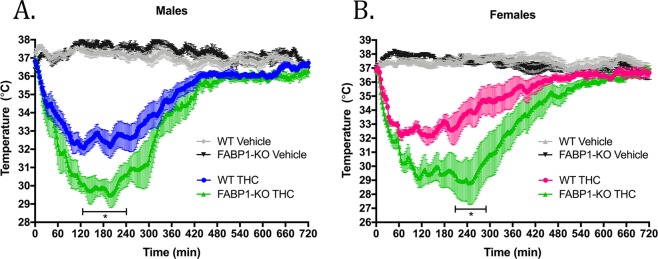
Time-course of THC-induced hypothermia. (**A**) Male and (**B**) female wild-type or FABP1-KO mice were treated with vehicle or 10 mg/kg THC. Bar indicates range where statistical significance was reached comparing the THC-treated groups. *p < 0.05 by two-way ANOVA with Bonferroni’s post-hoc test. n = 5–9 animals per group.

As expected, THC administration produced a rapid and significant drop in body temperature compared to the vehicle groups (Fig. 6). No significant difference in core temperature between WT and FABP1-KO animals was observed following injection of vehicle (Fig. 6). Both male and female FABP1-KO mice were found to exhibit a significantly potentiated magnitude and duration of hypothermic response to THC compared to their WT counterparts (Fig. 6A,B). We observed a maximum mean body temperature change of −4.8 °C in WT and −7.4 °C in FABP1-KO males, and −4.8 °C in WT and −8.1 °C in FABP1-KO females.

**Figure 7:**
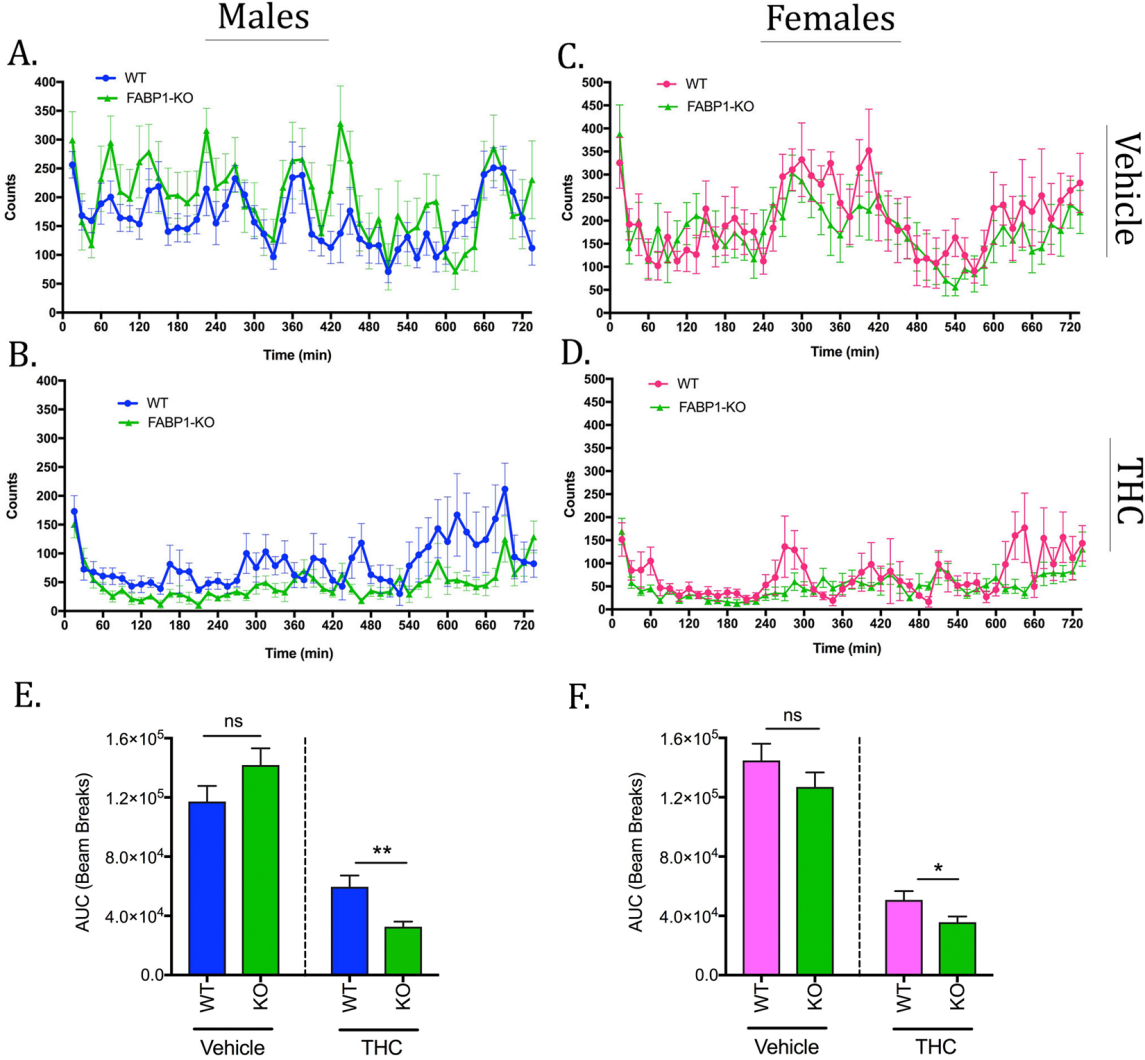
Home cage locomotor activity. Spontaneous motility in males (**A**,**B**) and females (**C**,**D**) over a 12 hour period following administration of vehicle (**A**,**C**) or 10 mg/kg THC (**B**,**D**). AUC analysis of the resulting motility curves in males (**E**) and females (**F**). *p < 0.05; **p < 0.01 by t-test. n = 9–12 animals per group.

In the spontaneous locomotor activity tests there were no significant differences observed between WT and FABP1-KO mice following injection of the vehicle (Fig. 7A,C). THC administration induced a significant decrease in overall motility in mice of both sexes (Fig. 7B,D). Recovery from THC-induced hypomotility was prolonged in FABP1-KO animals compared to wild-type mice (Fig. 7B,D). THC-treated FABP1-KO mice exhibited significantly decreased total motility compared to their WT counterparts over this time period irrespective of sex (Fig. 7E,F).

## Discussion

The hydrophobic nature of cannabinoids results in membrane sequestration and necessitates soluble carrier proteins for trafficking throughout the aqueous environments of the cytosol and extracellular fluids. Cannabinoids are transported by serum albumin and lipoproteins in the blood^39–41^, but until recently little was known about their mechanisms of intracellular transport. Over the past decade several members of the FABP family and other cytoplasmic lipid-solubilizing proteins, such as sterol carrier protein-2 and hsp70, have emerged as endo- and phyto-cannabinoid transport proteins within cells^10,16,42–46^. Efficacy of compounds like the endocannabinoid uptake inhibitor WOBE437 suggest that there remains yet unidentified proteins that also play roles in cellular cannabinoid transport^47^.

Our data confirm recent reports that FABP1 binds the phytocannabinoids THC and CBD with moderately high affinities, as well as the major THC metabolites 11-OH-THC and THC-COOH with comparatively reduced affinities^16,48^. The synthetic THC-COOH analog AJA was also found to interact with FABP1. The low micromolar affinities we observed *in vitro* may be physiologically relevant considering sequestration of these lipophilic compounds to membranes can result in very high subcellular local drug concentrations. Smoked or parentally administered cannabinoids rapidly distribute to highly perfused tissues and particularly accumulate within lungs and liver, suggesting that sufficient hepatic concentrations of these compounds may be reached *in vivo*^49^. High liver concentrations are also found following oral administration, as THC and CBD are subject to substantial first-pass effects^7^.

The newly solved crystal structure we report demonstrates that THC binds at only one site within the FABP1 protein. This crystal structure was utilized for *in silico* predictions of binding geometries and energetics of the non-psychoactive cannabinoids and THC metabolites. Computational analyses indicate that the observed reduced binding affinities of these compounds compared to the parent THC likely involves higher desolvation penalties incurred as a result of increased polarity. Despite the increased polar character, these THC metabolites are not themselves sufficiently hydrophilic for normal bodily excretion and likely also utilize protein-mediated shuttling to reach their respective intracellular metabolic enzymes.

We observed reduced rates of THC biotransformation in primary hepatocytes derived from FABP1-KO mice, suggesting a functional significance of FABP1 within the liver. However, it is noteworthy that another group recently reported an overall increase in THC metabolites found within THC-treated FABP1-KO hepatocytes^48^. This apparent discrepancy may potentially be explained through methodological differences in the hepatocyte culture confluency used for experiments, or in the methods of lipid extraction and quantification of metabolites. In our hands FABP1-KO hepatocytes metabolized THC at a lower rate compared to wild-type cells. However, appreciable THC biotransformation was still observed in FABP1-KO cells suggesting the existence of other putative hepatic protein(s) with redundant functions as phytocannabinoid carriers. Other cytoplasmic lipid-binding proteins, such as sterol carrier protein-2 and FABP2, are possible candidate transporters due to their presence in liver and documented roles as aqueous carriers for diverse lipophilic ligands, including endocannabinoids^44,50^.

The importance of FABP1 as a THC carrier was further confirmed by our observation that FABP1-KO mice exhibited reduced THC metabolism and a concomitant enhancement of its behavioral effects. Interestingly, no significant differences in THC pharmacodynamics were observed between the genders. Assessment of THC pharmacokinetics in mice found that FABP1-KO animals exhibit reduced THC clearance rates, as evidenced by increased AUCs, lower THC-COOH/THC AUC ratios, and the observation that THC and its metabolites remained at detectable levels in tissue for longer periods of time. Together, these data suggest a central role for FABP1 in hepatic THC inactivation.

In addition to its key role in THC biotransformation, FABP1 may also serve as a previously unrecognized site of drug-drug-interactions. Numerous xenobiotics including fibrates, warfarin, diazepam, gemfibrozil, flurbiprofen, indoprofen, and diclofenac bind to FABP1 with comparable affinities to THC^15,18,51,52^. Oral doses of these common medications often exceed 100–1000 mg per day, resulting in high tissue concentrations of drug and further suggesting that these compounds may compete with phytocannabinoids for cellular uptake and lead to unpredictable pharmacological profiles. Competition for FABP-mediated cellular uptake may also explain, at least in part, how non-psychotropic cannabinoids like CBD exert their effects. CBD has been implicated as an inhibitor of hepatic drug metabolism^53,54^. When co-administered, CBD inhibits THC metabolism resulting in higher blood and brain levels of THC in rats^55,56^ and mice pretreated with large quantities of CBD show drastically increased brain THC levels^57^. CBD partially inhibited THC hydroxylation in a double-blind placebo-controlled human study^58^. These observations raise the possibility that CBD competes with THC for FABP-mediated cellular uptake, but competition at their shared metabolic enzymes is also likely.

Phytocannabinoids may also potentiate endocannabinoid signaling through competition for FABP-mediated uptake and subsequent metabolism. Incubation of primary mouse hepatocytes with THC induces significant endocannabinoid accumulation indicative of reduced endocannabinoid metabolism, but this effect is not seen in hepatocytes derived from FABP1-KO animals^21^. Administration of CBD to humans reportedly results in elevated serum levels of the endocannabinoid anandamide, a finding attributed by the authors to inhibition of its catabolic enzyme fatty acid-amide hydrolase (FAAH)^59^. CBD robustly inhibits mouse and rat FAAH, but recent findings from our group and others demonstrate that CBD does not inhibit human FAAH activity^10,60^. In light of the present work, an alternative interpretation of these data may instead be that CBD targets cellular transport proteins involved in endocannabinoid clearance. It is plausible that a partial mechanism of action for some of CBD’s reported therapeutic effects occurs by competition for liver and/or brain FABPs and subsequent potentiation of endocannabinoid signaling. In conclusion, this study ascribes a novel role for FABP1 as a key regulator of THC inactivation and positions this protein as a potential site of drug-drug-interactions in patients administered medical marijuana along with other medications. Further work will be required to ascertain the clinical significance of competition for cellular uptake between phytocannabinoids and other lipophilic drugs.

## Methods

### Chemicals

CBD, THC, 11-OH-THC, THC-COOH and AJA were supplied by the Drug Supply Program at the National Institute on Drug Abuse. Deuterated THC-d_3_, 11-OH-THC-d_3_, and THC-COOH-d_3_ were each purchased from Cerillient (Round Rock, TX, USA). 11-(Dansylamino)unadecanoic acid (DAUDA) was purchased from Cayman Chemical Company (Ann Arbor, MI, USA).

### Animals

8–18 week old C57Bl/6 mice, and FABP1-KO mice on a C57Bl/6 background were used for all experiments. The mice were group-housed (3 per cage) at room temperature and kept on a 12/12 hour light/dark cycle with *ad libitum* access to food and water. Animals were single-housed for experiments and allowed at least one week to habituate prior to beginning. All experiments were approved by Stony Brook Institutional Animal Care and Use Committee (IACUC #735227) in accordance with the National Institutes of Health Guide for the Care and Use of Laboratory Animals.

### Protein purification

Human FABP1 in a pNIC28-Bsa4 vector was obtained from Addgene (plasmid #42344; Addgene, Cambridge, MA, USA). Following sequence verification the construct was transformed into BL21(DE3) *Escherichia coli* for expression. The recombinant FABP1 protein was purified by nickel-affinity chromatography as described previously^43^. Residual endogenous bacterial lipids were stripped from the purified protein by incubation in a column of hydroxyalkoxypropyl-dextran (Sigma Chemical Co., St Louis, MO, USA) for 1 hour at 37 °C.

### Crystallization and structure determination

Purified FABP1 was diluted to 1 mg/ml in a stabilizing solution containing 150 mM NaCl and 20 mM Tris HCl (pH 8.5) supplemented with 350 μM THC, and incubated for 18 hours at 4 °C followed by concentration to 25 mg/ml. Crystals were grown in a solution of 1.4 M sodium malonate, 0.1 M Bis Tris (pH 6.8) and cryo-protected by soaking in 25% glucose for less than 1 min prior to flash freezing in liquid nitrogen. X-ray diffraction data were collected at the BL14-1 beamline of Stanford Synchrotron Radiation Lightsource with a crystal to detector distance of 280 mm, an oscillation of 0.5°, and an exposure time of 1 s. Crystals belonged to space group P2_1_2_1_2_1_ and diffracted to 2.5 Å resolution. Data were integrated and scaled using HKL200^61^. The structure was solved by molecular replacement using the structure of FABP1 bound to a single palmitic acid molecule as a search model (PDB: 3STM). Analysis of the reflection intensity distribution in PHENIX indicated the precense of crystal twinning with the twin law k,h,-l^62^. Structure refinement was carried out using PHENIX refined twin fraction of 0.49. Final data collection and refinement statistics are given in Table 1. The positions of bound THC molecules were confirmed by calculating simulated annealing OMIT maps in PHENIX. The models were validated using Molprobity^63^. The atomic coordinates and structure factors have been deposited in the PBD database (PDB: 6MP4).

### Docking calculation structure preparation

The protein was prepared for docking using the DOCK6 protocol as outlined in Allen *et al*.^28^. Briefly, the protein was protonated and assigned parm99^64^ force field parameters and partial charges using the *tleap* module distributed with the program AMBER^65^. The parent THC ligand was assigned the GAFF^66^ force field augmented with AM1-BCC^67,68^ atomic charges using *antechamber*^69^. The complex was then subjected to a short energy minimization with restraints on all heavy atoms to relax the system prior to docking. The minimized complex was then prepared for docking which entailed conversion of AMBER files to MOL2 format and generation of receptor molecular surfaces, spheres, and energy grids required for DOCK, as previously described^28^. Metabolites and analogs of THC were constructed by modifying the parent molecule starting from the crystallographic pose using the *build structure* function in the program Chimera^70^. As before, ligands were assigned AM1-BCC atomic charges. All ligands were then re-docked into FABP1 using DOCK’s fixed-anchor docking (FAD)^28^ protocol to sample chemical moieties that differed from the parent using DOCK6 and obtain grid-based DOCK energies. For comparison, single point MM-GBSA calculations were also performed on the docked poses, using the Hawkins *et al*.^29,30^ GB model as implemented into DOCK6, to assess the impact of solvation.

### Probe Kd determination

Recombinant human FABP1 (1 μM) was titrated with DAUDA (0–16 μM). The raw fluorescence intensity at each data point was corrected by subtracting the signal from each respective probe concentration in the absence of protein. K_d_ and B_max_ were then calculated by fitting the titration curve to the single-site saturation binding equation [Y = B_max_*X/(K_d_ + X)] using the GraphPad Prism software (Prism version 7.0 for Mac OS: Graphpad software Incorporated, La Jolla, CA, USA).

### Fluorescence displacement binding assays

Binding assays were carried out in 96-well Costar^®^ plates (Corning Life Science, Kennebunk, ME, USA). FABP1 (3 μM) was incubated with DAUDA (500 nM), in binding assay buffer (30 mM Tris-HCl, 100 mM NaCl, pH 7.6). Competitor test compounds (0.1–500 μM) were then introduced to the well, mixed, and the system was allowed to equilibrate for 20 minutes at 25 °C in the dark. All experimental conditions were tested in triplicate. Each independent assay also included a strong competitive binder (oleic acid, 10 μM) as a positive control for probe displacement, and also background reading wells that did not contain any protein. Loss of fluorescence intensity was monitored with an F5 Filtermax Multi-Mode Microplate Reader (Molecular Devices, Sunnyvale, CA, USA) using excitation/emission wavelengths = 345/535 nm. Following background subtraction, raw fluorescence intensity values were normalized and fit to a one-site competition binding analysis using the Graphpad Prism software.

### Surgery and body temperature measurements

Mice were anesthetized with an i.p. injection of a surgical mixture of ketamine (100 mg/kg) and xylazine (10 mg/kg). Thermocron iButton^®^ (Dallas Semiconductor Company, Dallas, TX, USA) temperature data loggers were sterilized with 70% ethanol and surgically implanted into the abdominal cavities. The mice were permitted to recover from surgery for at least one week prior to being subjected to any experiments. The factory calibrated thermocrons were programmed to record temperature measurements every three minutes with 0.5 °C accuracy and 0.0625 °C precision.

### Home-cage motility

Locomotor activity was measured by the home-cage photobeam activity system (San Diego Instruments, San Diego, CA, USA). In this system each mouse home-cage was surrounded by a 4 × 8 photobeam array that detected and recorded movements in the X and Y directions. The animals were first conditioned to the cages in the testing room for one week to avoid novelty induced changes in locomotor activity. THC or vehicle were injected 10 minutes prior to the onset of the dark cycle and motility data was recorded over the next 12 hour period.

### Pharmacokinetics

Mice were administered THC (10 mg/kg, i.p.) and plasma and brain samples were collected at the following time points: 0, 0.5, 1, 2, 3, 4, 6, 7, 24 hours. Tissues were immediately placed on dry ice and stored at −80 °C until processing. The AUC from time 0 to 24 h was calculated by the trapezoidal rule.

### LC-MS/MS quantification of THC and THC metabolites

The tissues were rapidly thawed, spiked with 25 μl deuterated internal standard solution (containing THC-d_3_, 11-OH-THC-d_3_, and THC-COOH-d_3_; 5 ng/mL each, in methanol) (Cerillient Corporation, Round Rock, TX, USA) and homogenized on ice in Tris, pH 8.0. Protein precipitation was performed by adding two volumes ice-cold acetonitrile drop-wise while vortexing, centrifuged at 10,000 RCF for 10 minutes at 4 °C, and stored overnight at −20 °C to allow time for complete phase separation. The organic phase was transferred to a new glass tube and dried down under a steady stream of argon gas. The dried samples were resuspended in 100 μl 1:1 acetonitrile:ddH_2_O and submitted to the Stony Brook Proteomics Center for LC-MS/MS quantification of THC and THC metabolites.

### Isolation and culture of primary mouse hepatocytes

Mice were euthanized by CO_2_ asphyxiation and their livers were rapidly excised and perfused with ‘perfusion buffer’ (10 mM HEPES in Ca^2+^/Mg^2+^-free HBSS, 1 mg/mL gentamycin sulfate, 0.5 mM EGTA, pH 7.4). The livers were subsequently perfused with ‘collagenase buffer’ (perfusion buffer without EGTA and supplemented with 5 mM CaCl_2_ + 100 units/mL collagenase type IV) and the livers gently palpated to facilitate release of hepatocytes. The hepatocytes were collected, washed three times with ice-cold DMEM/F12 + 5% FBS, viability assessed by trypan blue staining, and then seeded on to collagen-coated 6 cm culture plates at a density of 1 × 10^6^ cells/plate in hepatocyte culture media (DMEM/F12, 10 mM HEPES, 5% FBS, 100 units/mL gentamycin sulfate). The cells were cultured overnight in a CO_2_ incubator at 37 °C and were used for experiments the following day.

### THC biotransformation by cultured primary hepatocytes

Primary mouse hepatocytes were washed with warm HBSS and incubated with serum-free DMEM/F12 + 0.1% BSA + 10 µM THC for 5–60 minutes at 37 °C. THC was added in DMSO and the final solvent concentration was kept under 0.5%. At the indicated time-points the culture plates were placed on ice, cells scraped, and the media/cell mix were transferred to a new glass tube on ice. The reaction was immediately quenched by the addition of 2.5x volumes of ice-cold acetonitrile and the samples were processed for LC-MS/MS quantification of 11-OH-THC as described above.

### Microsome isolation and enzyme assays

Crude hepatic microsomes isolated were isolated from wild-type and FABP1-KO livers. Livers were homogenized in KCl-sucrose buffer (150 mM KCl, 25 mM sucrose in 50 mM phosphate buffer, pH 7.5) and centrifuged at 10,000 RCF for 25 minutes, then the supernatant spun at 105,000 RCF for 80 minutes at 4 °C with a SW-60 ultracentrifuge rotor. The supernatant was discarded and the pellet gently washed three times with phosphate buffer (50 mM phosphate buffer, pH 7.5). The pellets were resuspended in 80% phosphate buffer, 20% glycerol and homogenized thoroughly on ice with a glass dounce homogenizer. Microsomal protein content was determined by the BCA method with bovine serum albumin (BSA) as a standard. THC (130 μM final concentration, added in 3 μl DMSO) was added to 0.2 mg of crude microsomal protein or BSA control in potassium phosphate buffer (100 mM potassium phosphate, pH 7.4) and pre-incubated for 3 minutes on ice. NADPH (1 mM) was introduced to start the reaction and then was allowed to proceed for 10 minutes at 37 °C with shaking. 2.5 volumes of ice-cold acetonitrile was used to quench the reaction. The solution was spiked with deuterated THC, 11-OH-THC and THC-COOH standards and processed for phytocannabinoid quantification by LC-MS/MS as described above. Microsomal enzymatic activity was additionally assessed by luciferin-H substrate conversion using the CYP450-Glo™ CYP2C9 assay kit (#V8791; Promega, Madison, WI, USA) with 20 µg liver microsomes or BSA control, according to the manufacturer’s instructions. Reactions were carried out for 30 minutes at 37 °C in the presence or absence of the CYP2C9 inhibitor sulphaphenazole (30 µM).

### Statistical analysis

All quantitative data represent mean ± S.E.M. Unpaired two-tailed t-test or two-way ANOVA with Bonferroni’s post-hoc test were used, as appropriate, to determine significance between the means. Statistical analysis and data visualization were performed using the Graphpad Prism software.

## Supplementary information

Supplementary Information

## Data Availability

The datasets generated during this study are available from the corresponding authors upon request.
