# Supplementary material for: FABP1 controls hepatic transport and biotransformation of Δ9-THC
Source: Sci Rep. 2019 May 20;9:7588. doi: 10.1038/s41598-019-44108-3 (PMC6527858; doi:10.1038/s41598-019-44108-3)
Supplement: Supplementary file 1 — Supplementary Information [file 41598_2019_44108_MOESM1_ESM.pdf]

FABP1 controls hepatic transport and biotransformation of Δ^9-THC

Matthew W. Elmes[1,2,3,*], Lauren E. Prentis[4], Luke L. McGoldrick[1], Christopher J. Giuliano[1], Joseph M. Sweeney[1], Olivia M. Joseph[1], Joyce Che[1], Gregory S. Carbonetti[1,2,3], Keith Studholme[2], Dale G. Deutsch[1], Robert C. Rizzo[4], Steven E. Glynn[1], and Martin Kaczocha[1,2,*]

[1]Department of Biochemistry and Cell Biology, Stony Brook University, Stony Brook, New York 11794, USA.

[2]Department of Anesthesiology, Stony Brook University, Stony Brook, New York 11794, USA.

[3]Graduate Program in Molecular and Cellular Biology, Stony Brook University, Stony Brook, New York 11794, USA.

[4]Department of Applied Mathematics and Statistics, Stony Brook University, Stony Brook, New York 11794, USA

*Corresponding Author: Correspondence and requests for materials should be addressed to M.W.E. (matthew.elmes@stonybrook.edu) or M.K. (martin.kaczocha@stonybrook.edu).

Supplementary Information

Supplementary Table S1. Crystallographic data collection and refinement statistics.

Data Collection	
Space Group	$P2_12_12_1$
Unit Cell Parameters (Å)	a=79.2 b=79.3 c=234.6
Resolution (Å)	40.52-2.50 (2.54-2.50)
Measured Reflections	422700
Unique Reflections	51960
Completeness (%)	100.0 (100.0)
Multiplicity	8.1 (7.9)
$\langle I/\sigma I \rangle$	69.5 (1.9)
R_{merge}	0.089 (1.135)
R_{meas}	0.095 (1.214)
$CC_{1/2}$ of highest resolution shell	0.901
Refinement	
Resolution (Å)	40.38-2.50
R_{work}/R_{free} (%)	20.6/22.5
No. of protein atoms	8634
No. of waters	305
No. of ligand atoms	90
R.m.s.d. bond lengths (Å)	0.004
R.m.s.d. bond angles (°)	1.06
Mean *B* factor (Å^2)	64.84
Mean B factor for water (Å^2)	47.12
Mean B factor for ligands (Å^2)	60.78
Ramachandran Plot Statistics Favored/Allowed/Disallowed (%)	98.04/1.96/0.00
Molprobity Score	1.72

Supplementary Table S2. *In vitro* activities and computationally predicted interaction energies for cannabinoids and THC metabolites to human FABP1.

**Compound Code**	**Experimental K_i (μM)**	**Grid Score[a] (kcal/mol)**	**MM-GBSA Score[b] (kcal/mol)**	**ΔG_{HYD} Ligand[c] (kcal/mol)**
THC	2.93±0.27	-54.42	-25.33	-6.79
11-OH-THC	7.12±0.79	-57.29	-24.78	-14.04
THC-COOH	11.2±0.7	-62.87	-16.76	-85.67
AJA	26.0±3.8	-69.30	-8.70	-85.13
CBD	3.95±0.65	-58.28	-32.76	-9.22

[a]DOCK grid score = VDW + ES energy, [b]MM-GBSA score = VDW + ES energy + $\Delta\Delta G_{HYD}$, [c]ΔG_{HYD} Ligand = ΔG_{POLAR} + $\Delta G_{NONPOLAR}$.

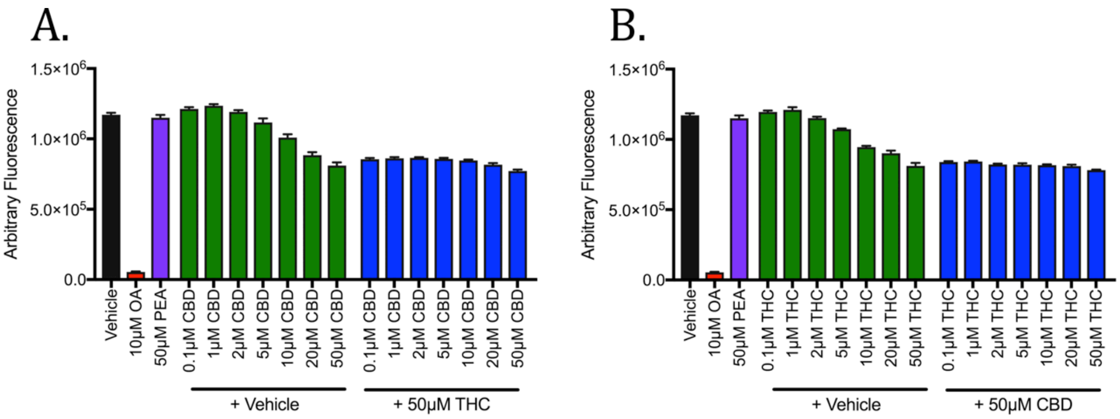

Supplementary Figure S1. FABP1 competitive binding assays. (**A**) DAUDA displacement from FABP binding pocket by CBD (0.1-50 μM) in the presence or absence of 50 μM THC. (**B**) DAUDA displacement by THC (0.1-50 μM) in presence or absence of 50 μM CBD. Assays include positive (oleic acid, 10 μM) and negative (palmitoylethanolamide, 50 μM) controls for probe displacement.

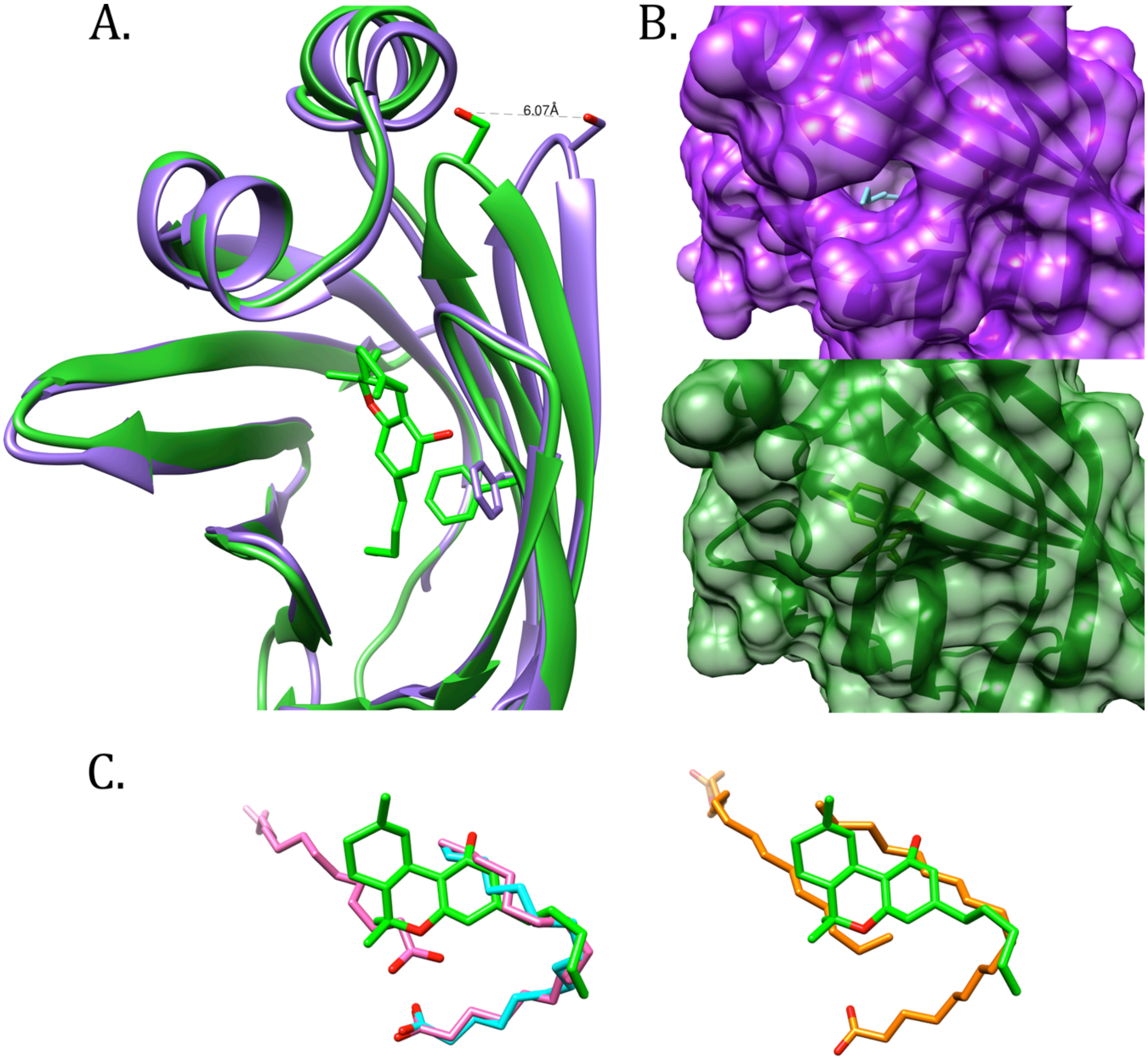

Supplementary Figure S2. Structure overlay between THC-bound FABP1 (PDB: 6MP4, green) and palmitic acid-bound FABP1 (PDB: 3STM, purple). (**A**) Large 6 Å movement of the β3/β4 β-turn is observed in the THC-bound structure. (**B**) Surface views of 3STM and 6MP4 at identical angles depicts the FABP1 ligand entry portal closure when in complex with THC. (**C**) Comparison of THC (green) ligand binding pose with published palmitic acid-bound (left; blue, PDB: 3STM; pink, PDB: 3STK) and oleate-bound (right; orange, PDB: 2LKK) human FABP1 structures.

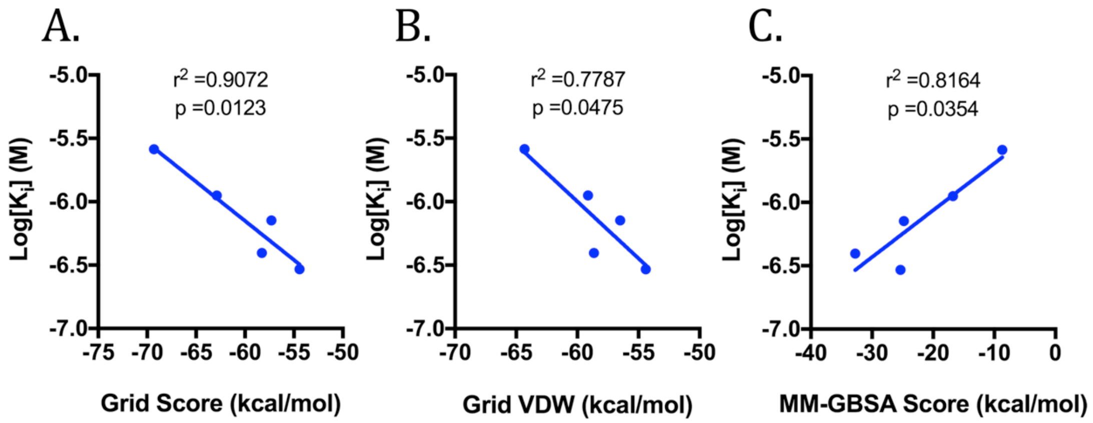

Supplementary Figure S3. Correlation analyses of computational predictions to experimentally determined affinities. (**A**) Grid score, (**B**) Grid van der Waals score, and (**C**) MM-GBSA score that includes desolvation penalties.

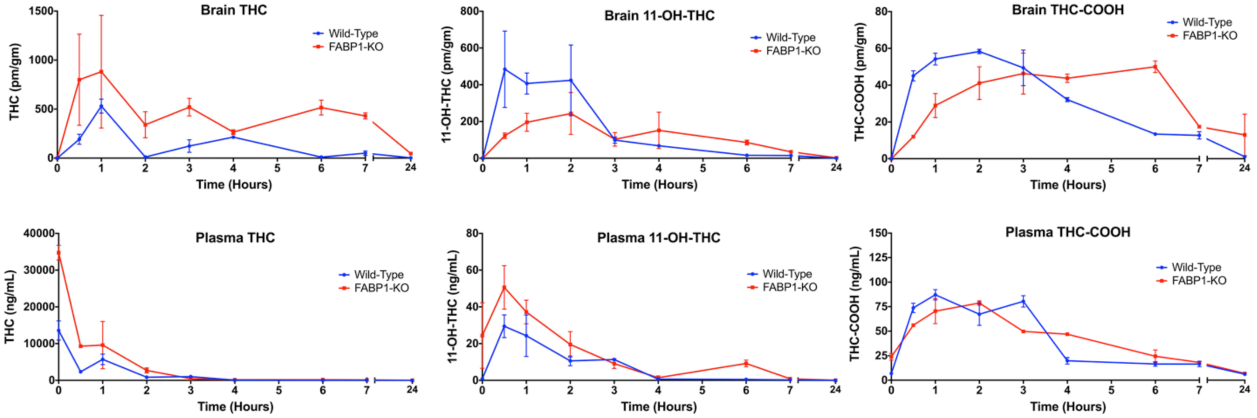

Supplementary Figure S4. THC pharmacokinetics in wild-type and FABP1-KO male mice. Brain (**A**-**C**) and plasma (**D**-**F**) levels of THC (**A**,**D**), primary THC metabolite 11-OH-THC (**B**,**E**), and secondary metabolite THC-COOH (**C**,**F**), following administration of THC (10 mg/kg, i.p.). n = 4 animals per group.
